# Association of distal adenoma and hyperplastic polyp characteristics with long-term proximal colon cancer risk: a secondary, observational analysis of data from the UK Flexible Sigmoidoscopy Screening Trial

**DOI:** 10.1136/bmjgast-2025-001787

**Published:** 2025-06-23

**Authors:** Rhea Harewood, Kate Wooldrage, Emma C Robbins, James Kinross, Christian von Wagner, Amanda J Cross

**Affiliations:** 1 Cancer Screening and Prevention Research Group (CSPRG), Department of Surgery and Cancer, Imperial College London, London, UK; 2 Department of Surgery and Cancer, Imperial College London, London, UK; 3 Research Department of Behavioural Science and Health, University College London, London, UK

**Keywords:** COLORECTAL CANCER, ENDOSCOPY, COLORECTAL ADENOMAS, COLONIC POLYPS, COLORECTAL CANCER SCREENING

## Abstract

**ABSTRACT:**

**Objectives:**

Colorectal cancer screening with flexible sigmoidoscopy focuses on the distal colorectum, but it is unclear which distal polyp characteristics are associated with future proximal colon cancer incidence. We examined associations between distal adenoma or hyperplastic polyp characteristics and long-term incident proximal colon cancer.

**Methods:**

In secondary, observational analyses of UK Flexible Sigmoidoscopy Screening Trial data, we obtained data on the number and size of distal hyperplastic polyps (n=4872) and adenomas (n=4581), adenoma histology and dysplasia from endoscopy and pathology reports for screened asymptomatic participants. Adjusted HRs and 95% CIs for the association between distal polyp characteristics and proximal colon cancer incidence were estimated using multivariable Cox proportional hazard models.

**Results:**

Over a median of 20.7 years of follow-up (IQR 16.5–21.7), 110 proximal colon cancers were diagnosed among participants with distal adenomas and 96 were diagnosed among those with only distal hyperplastic polyps detected at baseline. Larger adenoma size (6–9 mm vs ≤5 mm: HR 1.67 (95%CI: 1.07 to 2.59) and ≥10 mm vs ≤5 mm: HR 2.08 (95%CI: 0.98 to 4.43); p=0.037) and high-grade (vs low-grade) adenoma dysplasia (HR 2.82, 95% CI: 1.34 to 5.93; p=0.012) at baseline were positively associated with proximal colon cancer incidence. No associations were observed for distal adenoma number overall or histology, or the number or size of hyperplastic polyps and proximal colon cancer incidence.

**Conclusions:**

We found some evidence that larger distal adenomas and those with high-grade dysplasia at baseline were positively associated with proximal colon cancer incidence. Larger studies are needed to confirm these findings.

**Trial registration number:**

ISRCTN28352761.

**WHAT IS ALREADY KNOWN ON THIS TOPIC**

- Previous studies (using flexible sigmoidoscopy trial data) have reported associations between baseline distal adenoma number and size and synchronous baseline proximal adenomas and between baseline distal adenoma number, size and histology and synchronous baseline advanced proximal neoplasia.
- To our knowledge, no previous study has investigated the association between distal polyp findings and long-term proximal colon cancer risk.

**WHAT THIS STUDY ADDS**

- We examined if distal findings were associated with long-term (21 years of follow-up) proximal colon cancer incidence among asymptomatic individuals with distal polyps detected at baseline screening flexible sigmoidoscopy.
- We found that distal adenoma size (>5 mm vs ≤5 mm) and distal adenoma dysplasia (high-grade vs low-grade) were associated with a higher risk of proximal colon cancer during follow-up.

**HOW THIS STUDY MIGHT AFFECT RESEARCH, PRACTICE OR POLICY**

- This study provides evidence on associations between distal endoscopic findings and future proximal colon cancer risk to inform both the need for colonoscopy and postpolypectomy guidelines to reduce proximal colon cancer risk.

## Introduction

 Colorectal cancer was estimated to be the third most common cancer and second most common cause of cancer death globally in 2022.^1 2^ Endoscopic screening can reduce colorectal cancer incidence by the removal of precursor lesions. Compared with colonoscopy, flexible sigmoidoscopy is a less expensive and less resource-intensive test and is associated with fewer adverse side-effects, examination-related complications and patient discomfort.^3 4^ However, unlike a colonoscopy, which examines the entire colorectum, flexible sigmoidoscopy only reaches the distal one-third of the colorectum and not the proximal region.^5^

Four randomised controlled trials (RCTs) within Europe and the USA examined the effectiveness of flexible sigmoidoscopy, used as a first-line screening test, at reducing long-term colorectal cancer incidence and mortality.^6^,^9^ During these trials, individuals found to have polyps categorised as ‘high-risk’ were referred for colonoscopy to examine the entire colorectum. However, despite evidence of reductions in distal colorectal cancer incidence, there was no evidence of an impact on long-term proximal colon cancer incidence.^6^,^9^

Studies have shown that individuals with distal polyps are more likely to have synchronous proximal neoplasia present compared with those with no polyps.^10 11^ With respect to future proximal colon cancer incidence, one secondary analysis using UK Flexible Sigmoidoscopy Screening Trial (UKFSST) data found that having low-risk distal polyps (1–2 tubular adenomas <10 mm with low-grade dysplasia and/or hyperplastic polyps<10 mm) was associated with a greater risk of proximal colon cancer compared with having no polyps.^12^ However, some polyp characteristics confer a greater risk of future cancer compared with others.^13^ Therefore, to inform postpolypectomy surveillance guidelines, it is necessary to identify which distal polyp characteristics are associated with proximal colon cancer incidence among those with distal polyps.

This study examined the association between distal polyp characteristics and long-term incidence of proximal colon cancer among asymptomatic individuals with polyps detected during flexible sigmoidoscopy screening.

## Methods

### Study design and population

The UKFSST is a large-scale RCT (ISRCTN28352761)^14^ that recruited individuals 55–64 years of age and registered with one of 506 general practitioners across 14 UK centres between 1994–1999.^15^ Details on the study design and methods have been previously reported.^15^,^18^ In summary, a total of 170 432 people were randomised (1:2) with 57 237 assigned to the flexible sigmoidoscopy screening intervention group and 113 195 to the control group (no screening, the standard care at the time). A total of 40 639 participants attended screening. Participants suspected to have ‘high-risk’ polyp findings (polyps ≥10 mm, ≥3 adenomas, adenomas with tubulovillous or villous histology, adenomas with high-grade dysplasia, malignancy or ≥20 hyperplastic polyps above the distal rectum) were referred for colonoscopy and/or surgery.^16^ Participants confirmed to have ‘high-risk’ findings were referred for colonoscopy surveillance.^9^

This current study included secondary, observational analyses using data from screened UKFSST participants. From the 40 639 screened participants, we excluded participants with prevalent cancer at baseline or colorectal cancer diagnosed within 11 months of baseline, those initially screened with colonoscopy rather than flexible sigmoidoscopy, those screened during the initial months at one centre where the pathologist was found to be over-diagnosing adenomas,^17^ those with a history of colectomy, possible inflammatory bowel disease, polyposis or hereditary colorectal cancer syndrome at baseline or those with an extended baseline visit (>11 months). As this study focused on associations with proximal colon cancer following distal colorectal polypectomy during flexible sigmoidoscopy, participants with no distal polyps detected at the baseline screening were also excluded.

### Exposures at baseline endoscopy

For this analysis, distal polyps were grouped as hyperplastic polyps (including sessile serrated lesions (SSLs) as no distinction was made between these two types of lesions at the time of the UKFSST) or adenomas. Baseline data were collected on the number and size of hyperplastic polyps and adenomas detected and on the histology and dysplasia of adenomas, as characterised using WHO criteria.^19^ Baseline participant and examination characteristics, including whether participants attended a non-flexible sigmoidoscopy examination (colonoscopy and/or surgery), were also collected.

A baseline visit included the first and any subsequent flexible sigmoidoscopy examinations as well as any colonoscopies or surgery performed following endoscopist referral. Baseline distal polyp/adenoma characteristics and data on examination completeness and quality were defined for each participant using information from all baseline examinations (including colonoscopy and/or surgery following flexible sigmoidoscopy) by assigning polyp size as the largest reported, adenoma histology as the most villous, grade as the highest grade of dysplasia, and completeness of examination (to the junction of the sigmoid and descending colon) and bowel preparation quality as the most complete or highest quality, respectively. For participants offered a colonoscopy or surgery, only data on polyps detected in the distal colorectum (between the rectum and the sigmoid colon) were included to define distal polyp characteristics.

Data on subsequent endoscopic surveillance at trial hospitals were collected through 2012 for screened participants who were referred for surveillance in the trial but not for those discharged at baseline. A surveillance visit was defined as a flexible sigmoidoscopy or colonoscopy examination (excluding those examinations where a referral for symptoms was reported) as well as any subsequent follow-up examinations performed within approximately 11 months.

### Outcome

Data on colorectal cancer incidence and on deaths were obtained from national cancer registries, National Health Service (NHS) Central Register, NHS Digital (now NHS England), National Services Scotland and the Office for National Statistics up to 31 December 2017 in Wales, 31 December 2018 in Scotland and 30 September 2019 in England.^9^ Under the National Data Opt-Out programme, which allows participants to opt out of having their data used for research, death and cancer data were unavailable for some participants in England in this current analysis.^9^ The outcome of interest was proximal colon cancer, defined as those located between the caecum and the descending colon (beyond the reach of the flexible sigmoidoscope) (International Classification of Diseases tenth edition (ICD-10) codes^20^ C18.0–C18.6). International Classification of Diseases for Oncology second edition (ICD-O-2)^21^ morphology codes were used to identify cases of invasive adenocarcinoma, and morphology codes for non-specified carcinoma were included where cancer was confirmed by clinical examination only.^15^

### Statistical analyses

Separate analyses were conducted on participants with distal adenomas at baseline, either alone or along with distal hyperplastic polyps, and on those with distal hyperplastic polyps only. The distribution of polyp characteristics at baseline and of cancer diagnosed throughout follow-up was described by various demographic, examination and surveillance factors.

Follow-up time began at the date of the latest baseline examination and ended at the earliest date of colorectal cancer incidence at any subsite, death, emigration or end of follow-up (those who opted out under the National Data Opt-Out programme were censored at end of follow-up as it was not possible to identify these participants).^9^ In four participants (two with only hyperplastic polyps and two with adenomas detected at baseline), colorectal cancer was diagnosed in both the proximal colon and the distal colon (three participants had proximal colon cancer diagnosed first and one had both cancers diagnosed on the same day); these cases were defined as proximal colon cancer.

Proximal colon cancer incidence rates per 100 000 person-years were calculated. Cumulative proximal colon cancer incidence probabilities over 20 years were calculated using the Kaplan-Meier method and were compared between exposure subgroups using the log-rank test. Crude HRs and 95% CIs for each polyp characteristic were estimated using Cox proportional hazard models, with the incidence of proximal colon cancer as the outcome and follow-up time as the underlying time metric. Statistically significant differences in HRs between exposure groups were assessed using the likelihood ratio test.

Multivariable analyses were conducted adjusting for a priori confounders, including sex, age, examination completeness and bowel preparation quality. For the adenoma analyses only, additional adjustments were made for the presence of distal hyperplastic polyps, attendance at a non-flexible sigmoidoscopy examination (ie, colonoscopy and/or surgery) during the baseline visit and number of surveillance visits attended included as a time-varying covariate (these variables were not included in the hyperplastic polyp analyses as the majority were not referred for additional examinations or for surveillance).

All statistical tests were two-sided and p values <0.05 were considered statistically significant. All analyses were conducted using Stata V.18.^22^

This study was reported according to the Strengthening the Reporting of Observational Studies in Epidemiology (STROBE) guidelines (online supplemental material).

## Results

### Baseline characteristics

Among 39 539 screened eligible participants, distal polyps were detected in 9453 individuals at baseline. Distal adenomas, either solely or in addition to distal hyperplastic polyps, were detected in 4581 participants and distal hyperplastic polyps alone were detected in 4872 participants (online supplemental figure 1).

Participants with any distal adenomas detected were followed for a median of 20.7 years (IQR 16.3–21.6 years), during which 110 proximal colon cancers were diagnosed. During a median follow-up of 20.7 years (IQR 16.7–21.7 years), 96 proximal colon cancers developed among participants with only distal hyperplastic polyps at baseline. The distribution of polyp findings, polyp characteristics and diagnosed cancers by demographic, examination and surveillance factors among participants with distal adenomas detected or those with only distal hyperplastic polyps detected are described in table 1 and online supplemental tables 1 and 2, respectively. Participants with ‘high-risk’ adenoma findings at baseline were less likely to have an incomplete examination and more likely to have a longer (>1 day) baseline visit. Those with ≥3 distal adenomas detected at baseline were more likely to also have distal hyperplastic polyps detected (online supplemental table 1).

**Table 1:** Description of participant demographics, examinations and surveillance visits by baseline distal polyp findings*

	All participants with distal polyps	Participants with distal adenomas	Participants with distal hyperplastic polyps only
N	9453	4581	4872
Sex			
Women	3473 (36.7%)	1526 (33.3%)	1947 (40.0%)
Men	5980 (63.3%)	3055 (66.7%)	2925 (60.0%)
Age group at randomisation (years)			
54–59	4617 (48.8%)	2168 (47.3%)	2449 (50.3%)
60–66	4836 (51.2%)	2413 (52.7%)	2423 (49.7%)
Completeness of examination†			
Complete	8755 (92.6%)	4357 (95.1%)	4398 (90.3%)
Incomplete	609 (6.4%)	194 (4.2%)	415 (8.5%)
Unknown	89 (0.9%)	30 (0.7%)	59 (1.2%)
Bowel preparation quality†			
Excellent or good	7269 (76.9%)	3716 (81.1%)	3553 (72.9%)
Adequate	1726 (18.3%)	671 (14.6%)	1055 (21.7%)
Poor	92 (1.0%)	39 (0.9%)	53 (1.1%)
Unknown	366 (3.9%)	155 (3.4%)	211 (4.3%)
Duration of baseline exam			
1 day	7509 (79.4%)	2904 (63.4%)	4605 (94.5%)
2–90 days	1426 (15.1%)	1210 (26.4%)	216 (4.4%)
91–334 days	518 (5.5%)	467 (10.2%)	51 (1.0%)
Had a colonoscopy and/or surgery during baseline following flexible sigmoidoscopy			
Yes	1620 (17.1%)	1519 (33.2%)	101 (2.1%)
No	7833 (82.9%)	3062 (66.8%)	4771 (97.9%)
Number of surveillance visits attended			
0	8171 (86.4%)	3339 (72.9%)	4832 (99.2%)
1‡	442 (4.7%)	421 (9.2%)	21 (0.4%)
≥2§¶	840 (8.9%)	821 (17.9%)	19 (0.4%)
Distal hyperplastic polyps			
No	3356 (35.5%)	3356 (73.3%)	–
Yes	6097 (64.5%)	1225 (26.7%)	–

* Baseline distal adenoma and hyperplastic polyp findings based on data from baseline flexible sigmoidoscopy examinations and data obtained on polyps detected between the rectum and the sigmoid colon during subsequent colonoscopy and surgery performed during a baseline visit.

† Completeness of examination and bowel preparation quality were defined using data from all examinations occurring during the baseline visit, that is, flexible sigmoidoscopy examinations and, where performed, subsequent colonoscopy and surgery during baseline.

‡ For participants with distal adenomas, the mean interval between baseline and the first surveillance visit was 2.8 years (SD 1.2 years); for participants with distal hyperplastic polyps only, the mean interval between baseline and the first surveillance visit was 3.0 years (SD 1.4 years).

§ For participants with distal adenomas, the mean interval between the first and second surveillance visits and the second and third surveillance visits were 3.6 years (SD 1.3 years) and 3.5 years (SD 1.4 years), respectively; for participants with distal hyperplastic polyps only, the mean interval between the first and second surveillance visits and the second and third surveillance visits was 3.3 years (SD 1.4 years) and 3.1 years (SD 1.2 years), respectively.

¶ For participants with distal adenomas, 11.3% had two surveillance visits, 4.9% had three surveillance visits and 1.7% had four or more surveillance visits.

Among participants with distal adenomas detected at baseline, 1519 (33%) had a colonoscopy and/or surgery during baseline following flexible sigmoidoscopy (1385 of whom had high-risk adenoma findings at baseline); this included >95% of participants with ≥3 adenomas, large adenomas (≥10 mm) or adenomas with tubulovillous or villous histology or high-grade dysplasia detected distally. Of those 1385 participants with any ‘high-risk’ distal adenoma finding at baseline who underwent a colonoscopy, proximal hyperplastic polyps and/or adenomas were detected in 22% (19% with adenomas) and 6% had at least one ‘high-risk’ proximal adenoma finding (online supplemental table 3).

Following baseline examination, 1242 (27%) participants with distal adenomas had at least one surveillance colonoscopy, with most participants with high-risk distal adenoma findings at baseline attending (79% of those with tubulovillous/villous distal adenomas detected at baseline up to 83% of those with high-grade dysplasia at baseline) (table 1; online supplemental table 1). Participants with only distal hyperplastic polyps generally did not have non-flexible sigmoidoscopy baseline examinations, with 101 (2%) having a colonoscopy and/or surgery during baseline post flexible sigmoidoscopy. Furthermore, a very small proportion of participants with only baseline distal hyperplastic polyps (0.8%) attended a surveillance colonoscopy visit (table 1).

### Participants with distal adenomas at baseline

**Figure 1:**
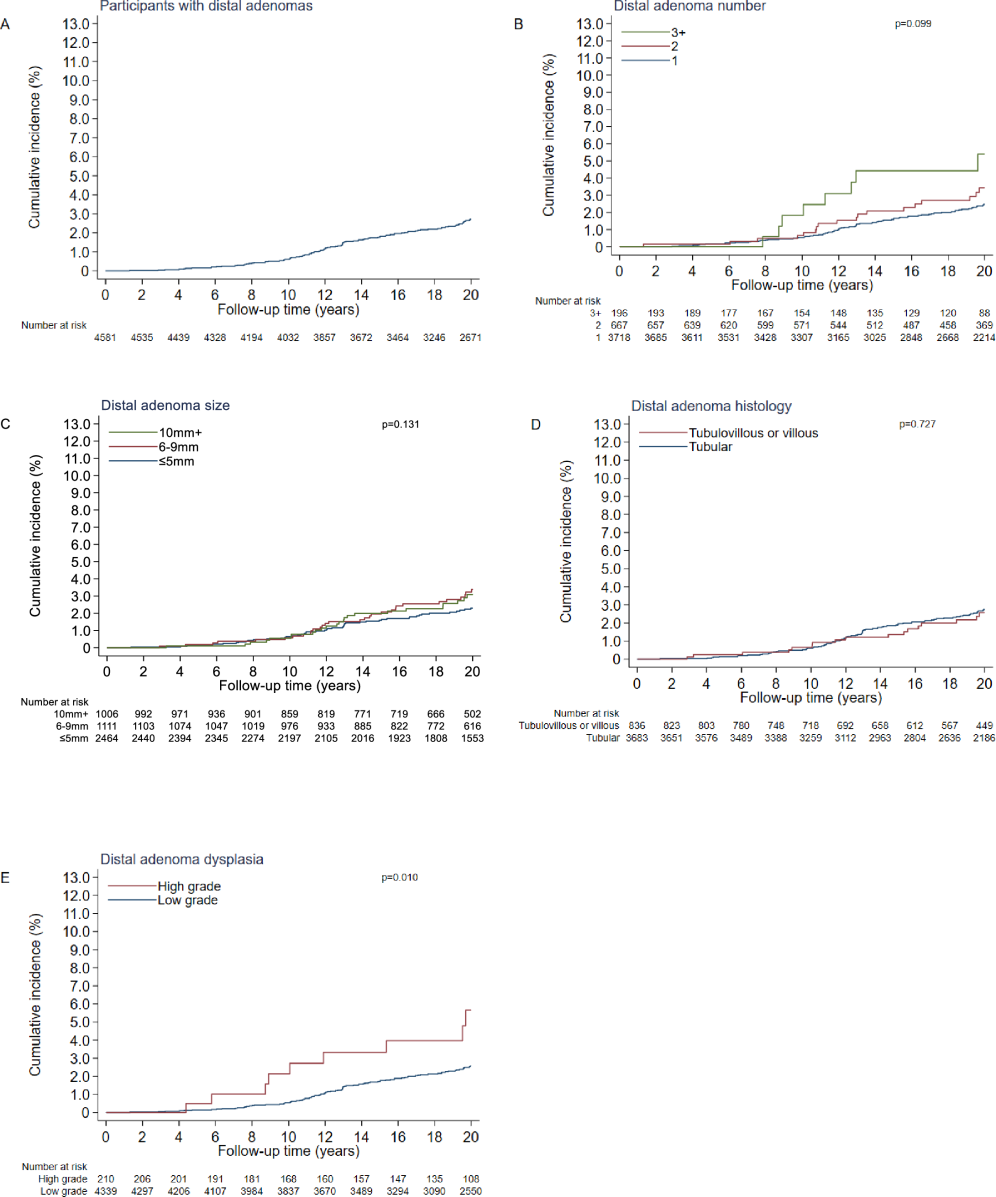
Cumulative incidence curves (%) for proximal colon cancer: overall (**A**); by number of distal adenomas (**B**); by size of the largest distal adenoma(s) in mm (**C**); by worst histology of distal adenoma(s) (**D**); by most advanced distal adenoma dysplasia (**E**). Participants with unknown values for a given adenoma characteristic are not displayed in the respective graph. P values calculated with the log-rank test comparing curves up to 20 years of follow-up.

Among the 4581 participants with any distal adenoma (alone or in addition to hyperplastic polyps) detected at baseline, the incidence rate of proximal colon cancer during follow-up was 133 (95% CI: 110 to 160) per 100 000 person-years and cumulative incidence at 20 years was 2.7% (95% CI: 2.2% to 3.3%) (table 2). Cumulative incidence at 20 years was higher among participants with high-grade compared with low-grade dysplasia (p=0.010) but was similar between all other subgroups of adenoma characteristics (table 2, figure 1).

**Table 2:** HRs and 95% CIs for proximal colon cancer incidence by baseline distal adenoma characteristics among participants with at least one distal adenoma at baseline

Baseline characteristics	Proximal colon cancer cases	Incidence rate per 100 000 (95% CI)	Cumulative incidence, % (95% CI)*	P value†	Univariable HR (95% CI)	P value‡	Multivariable HR (95% CI)§	P value‡
All participants with ≥1 distal adenoma(s)	110	133 (110 to 160)	2.7 (2.2 to 3.3)	–	–		–	
Number of distal adenomas				0.099		0.150		0.129
1	84	123 (100 to 153)	2.5 (2.0 to 3.1)	–	1.00	–	1.00	–
2	18	153 (97 to 243)	3.4 (2.2 to 5.4)	–	1.26(0.76 to 2.09)	–	1.27(0.75 to 2.14)	–
≥3	8	248 (124 to 495)	5.4 (2.7 to 10.6)	–	2.11(1.02 to 4.35)	–	2.40(1.06 to 5.42)	–
Distal adenoma size (mm)				0.131		0.138		0.037
≤5	51	112 (85 to 147)	2.3 (1.7 to 3.0)	–	1.00	–	1.00	–
6–9	34	171 (122 to 239)	3.4 (2.4 to 4.8)	–	1.54(1.00 to 2.38)	–	1.67(1.07 to 2.59)	–
≥10	25	143 (96 to 211)	3.1 (2.1 to 4.6)	–	1.31(0.81 to 2.11)	–	2.08(0.98 to 4.43)	–
Distal adenoma histology				0.727		0.725		0.950
Tubular	91	136 (110 to 166)	2.8 (2.2 to 3.4)	–	1.00	–	1.00	–
Tubulovillous or villous	18	122 (77 to 194)	2.6 (1.6 to 4.2)	–	0.91(0.55 to 1.52)	–	1.02(0.52 to 2.00)	–
Unknown¶	1	–	–	–	–	–	to	–
Distal adenoma dysplasia				0.010		0.026		0.012
Low grade	99	126 (103 to 153)	2.6 (2.1 to 3.2)	–	1.00	–	1.00	–
High grade	10	279 (150 to 519)	5.7 (2.9 to 10.7)	–	2.28(1.19 to 4.38)	–	2.82(1.34 to 5.93)	–
Unknown¶	1	–	–	–	–		–	–

* At 20 years of follow-up.

† P values calculated with the log-rank test comparing curves up to 20 years of follow-up.

‡ P values calculated with the likelihood ratio test.

§ Adjusted for sex, age, examination completeness, bowel preparation quality, presence of distal hyperplastic polyps, having had colonoscopy and/or surgery during baseline post flexible sigmoidoscopy and number of surveillance visits (0, 1 or ≥2; included as a time-varying covariate).

¶ Patients with unknown adenoma histology or grade were removed from incidence analyses, including adjusted models, as for both only one event was observed.

In multivariable adjusted models, larger adenoma size (6–9 mm vs ≤5 mm: HR 1.67 (95%CI: 1.07 to 2.59) and ≥10 mm vs ≤5 mm: HR 2.08 (95% CI: 0.98 to 4.43); p=0.037) and a higher grade of adenoma dysplasia (high grade vs low grade: HR 2.82 (95%CI: 1.34 to 5.93; p=0.012) were positively associated with proximal colon cancer incidence (table 2). However, the number of cases with ≥10 mm adenomas or adenomas with high-grade dysplasia was small (n=25 and n=10, respectively) and CIs for these HRs were wide. No association was observed for distal adenoma number overall or histology and proximal colon cancer (table 2), although those with ≥3 distal adenomas detected at baseline (compared with 1) had an increased risk (HR 2.40 (95% CI:1.06 to 5.42)); however, the number of proximal colon cancer cases was small in this category (n=8).

### Participants with distal hyperplastic polyps at baseline

**Figure 2:**
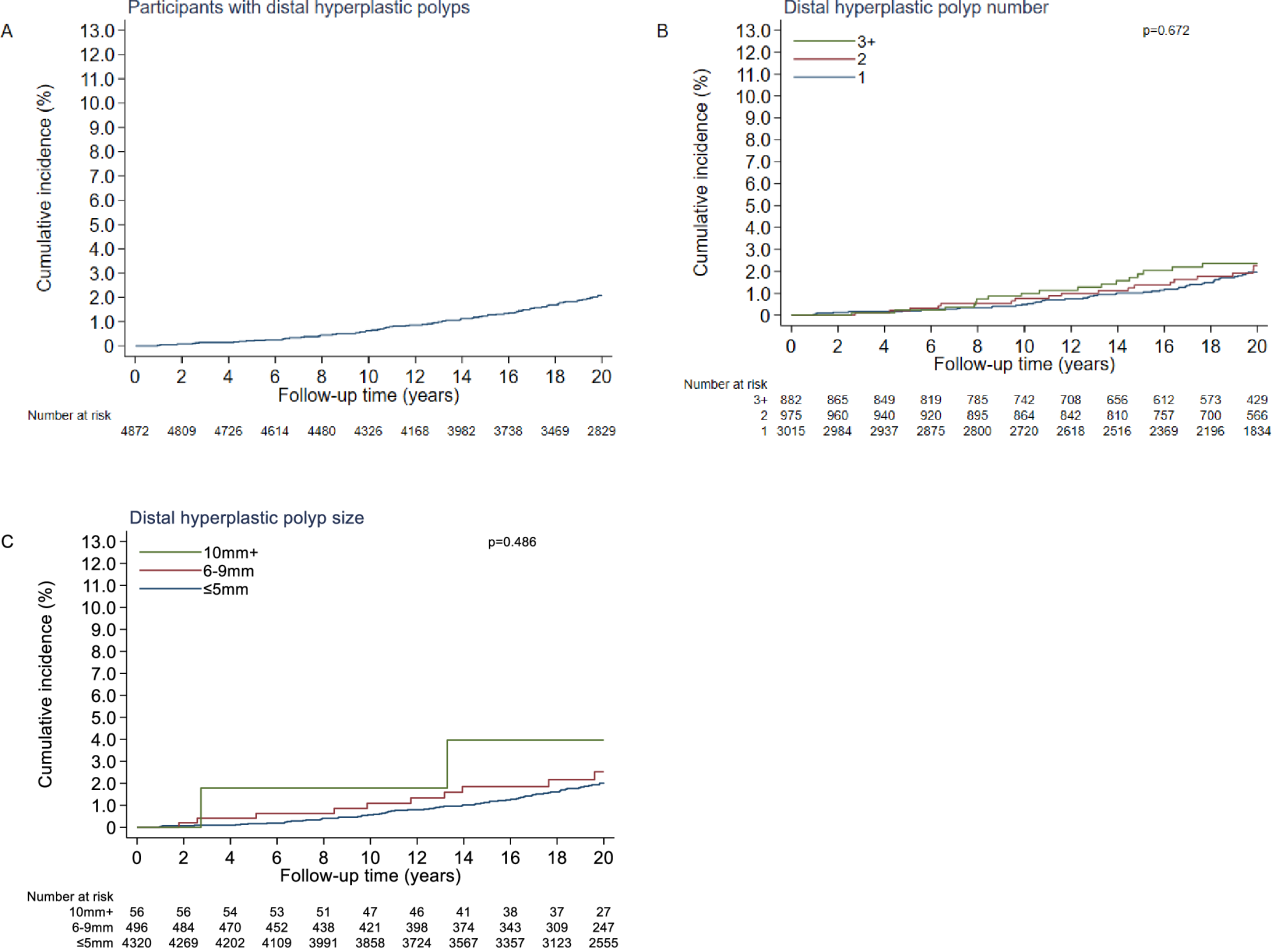
Cumulative incidence curves (%) for proximal colon cancer: overall (**A**); by number of distal hyperplastic polyps (**B**); by size of the largest distal hyperplastic polyp(s) in mm (**C**). P values calculated with the log-rank test comparing curves up to 20 years of follow-up.

Distal hyperplastic polyps alone were detected at baseline among 4872 participants, for whom the incidence rate of proximal colon cancer was 108 (95% CI: 88 to 131) per 100 000 person-years and cumulative incidence at 20 years was 2.1% (95% CI: 1.7% to 2.6%) (table 3). Cumulative incidence at 20 years was similar when comparing strata of distal hyperplastic polyp number and size (table 3, figure 2).

**Table 3:** HRs and 95% CIs for proximal colon cancer incidence by baseline distal hyperplastic polyp characteristics among participants with only distal hyperplastic polyps at baseline

Baseline characteristics	Proximal colon cancer cases	Incidence rateper 100 000 (95% CI)	Cumulative incidence, % (95% CI)*	P value†	Univariable HR (95% CI)	P value‡	Multivariable HR (95% CI)§	P value‡
Participants with distal hyperplastic polyps only	96	108 (88 to 131)	2.1 (1.7 to 2.6)	–	–		–	
Number of distal hyperplastic polyps				0.672		0.681		0.493
1	57	102 (78 to 132)	2.0 (1.5 to 2.6)	–	1.00	–	1.00	–
2	20	112 (72 to 174)	2.3 (1.4 to 3.6)	–	1.10(0.66 to 1.84)	–	1.17(0.70 to 1.95)	–
≥3	19	124 (79 to 195)	2.4 (1.5 to 3.8)	–	1.26(0.75 to 2.12)	–	1.37(0.81 to 2.31)	–
Distal hyperplastic polyp size (mm)				0.486		0.544		0.508
≤5	83	104 (84 to 129)	2.0 (1.6 to 2.5)	–	1.00	–	1.00	–
6–9	11	129 (71 to 232)	2.5 (1.4 to 4.7)	–	1.26(0.67 to 2.37)	–	1.30(0.69 to 2.45)	–
≥10	2	205 (51 to 820)	4.0 (1.0 to 15.1)	–	2.01(0.49 to 8.17)	–	2.01(0.49 to 8.21)	–

* At 20 years of follow-up.

† P values calculated with the log-rank test comparing curves up to 20 years of follow-up.

‡ P values calculated with the likelihood ratio test.

§ Adjusted for sex, age, examination completeness and bowel preparation quality.

Among participants with distal hyperplastic polyps only, there was no evidence of an association between distal hyperplastic polyp number or size and proximal colon cancer incidence; however, there were only two cases with hyperplastic polyps ≥10 mm in this analysis (table 3).

## Discussion

This study conducted in an asymptomatic screened population with polyps detected during flexible sigmoidoscopy screening found some evidence of a positive association between larger distal adenoma size and high-grade distal adenoma dysplasia and long-term incidence of proximal colon cancer. No association was observed between distal adenoma number overall, despite some evidence for an association comparing the highest and lowest categories, or histology, or distal hyperplastic polyp number or size, and the incidence of proximal colon cancer.

Previous meta-analyses in asymptomatic individuals have investigated the association between aggregated distal findings at baseline (ie, hyperplastic polyps, non-advanced or advanced adenomas) and synchronous proximal advanced neoplasia (advanced adenomas and/or cancer). These meta-analyses reported associations between distal adenomas and a higher odds of proximal advanced neoplasia, with odds increasing with increasing severity of the lesions compared with normal distal findings, but similar to this current study found no association with hyperplastic polyps.^10 11 23 24^ Analyses in another flexible sigmoidoscopy trial in Norway^25^ found positive associations between proximal advanced neoplasia at baseline and distal adenomas ≥10 mm, as for this current study, but also with multiple distal adenomas and distal adenomas with villous components (compared with adenomas <10 mm, one adenoma or adenomas without villous components, respectively). However, in these previous meta-analyses and trial analyses, outcome data were obtained at baseline representing prevalent proximal neoplasia. This current study is unique in its focus on long-term proximal colon cancer incidence with detailed data available on baseline polyp characteristics.

Among participants with small (6–9 mm) distal adenomas detected at baseline, most did not have a colonoscopy following flexible sigmoidoscopy or for surveillance purposes (72% and 78%, respectively), and most proximal cancers were diagnosed among those with no baseline colonoscopy (82%) or surveillance visits (88%). Post flexible sigmoidoscopy examination was even less likely among those with diminutive (≤5 mm) adenomas detected at baseline, and almost all proximal colon cancers were diagnosed among those with no baseline colonoscopy (49/51 cancers) or surveillance (50/51 cancers). The higher risk among those with small adenomas compared with diminutive ones could indicate they were more likely to have had proximal neoplasia at baseline and/or to develop it during follow-up. Coupled with evidence from previous analyses using UKFSST data of higher proximal colon cancer risk among women and men with small distal adenomas, respectively, compared with the general population,^12^ these findings warrant further investigation in larger cohorts and an examination of the potential benefits of colonoscopy for individuals with small distal adenomas.

A previous study conducted using data from the English Bowel Cancer Screening Programme (BCSP) found higher proportions of advanced histological features (high-grade dysplasia, tubulovillous/villous histology or carcinoma) present in adenomas with increasing size (3.5 and 15 times more likely among small (6–9 mm) and large (≥10 mm) adenomas, respectively, compared with diminutive (≤5 mm) adenomas).^26^ As adenomas are thought to progress to cancer via the adenoma-carcinoma pathway through stages of increasing adenoma size and levels of dysplasia,^27 28^ the presence of larger distal adenomas or adenomas with high-grade dysplasia could be indicative of more aggressive or faster growing polyps in those individuals. Furthermore, larger and more advanced polyps are more likely to be incompletely excised.^29 30^ These biological and examination factors could have increased the likelihood of interval cancers post colonoscopy or between surveillance visits.

This study benefited from the prospective design and long follow-up period. We used data from the UKFSST, the largest flexible sigmoidoscopy screening trial conducted within Europe, with over 40 000 participants receiving a flexible sigmoidoscopy screening examination. Most endoscopies were performed following standard protocols inferring a high level of accuracy and validity in the detection of polyps and reporting of characteristics. Detailed polyp data were collected allowing for examination of associations with individual polyp characteristics rather than just with aggregate polyp findings (eg, non-advanced or advanced adenomas). Algorithms were also used to ensure the process for summarising polyp findings and examination characteristics within each participant was standardised.

However, there are limitations. In this study, most participants (~96%) with at least one high-risk finding at flexible sigmoidoscopy had a follow-up colonoscopy and/or surgery at baseline. Of those with at least one ‘high-risk’ distal adenoma characteristic, up to 83% attended surveillance visits. These additional endoscopic examinations would have differentially impacted their future risk of proximal colon cancer compared with those participants with no exams post flexible sigmoidoscopy. During colonoscopy, the entire colorectum is examined, allowing for the detection and removal of additional hyperplastic polyps and adenomas in the proximal colon. Removal of these precancerous polyps could result in reductions in future proximal colon cancer risk compared with those participants with only ‘low-risk’ distal polyps detected who had no further examination of the entire colorectum or subsequent surveillance. The higher risk of proximal colon cancer observed among those with distal adenomas with high-grade dysplasia compared with those with low-grade dysplasia or distal adenomas ≥10 mm compared with those ≤5 mm in size (weaker evidence for the latter) in these current analyses is therefore an interesting finding. It may highlight a potential ineffectiveness of baseline and/or surveillance colonoscopy at reducing proximal colon cancer risk in individuals with such adenomas at baseline. In contrast, referral for colonoscopy among those with high-risk findings may have resulted in detection bias where they were more likely to have proximal colon cancer detected due to surveillance monitoring compared with those not referred for further examination. This latter scenario could have resulted in an overestimation of risk. The impact of this is likely to be minimal, as only one-quarter of cancers diagnosed through 2012 were detected at surveillance, and after 2012, when surveillance data were no longer available, few participants remained within the age criteria for surveillance. Statistical models were adjusted for colonoscopy and/or surgery at baseline and for surveillance visits, but there remains the possibility of residual confounding due to differential screening and surveillance patterns between participants with ‘high-risk’ compared with ‘low-risk’ distal polyps at baseline.

Surveillance data were not available after 2012, nor were any data on colonoscopies performed outside of trial centres or in the private sector (16% of all colonoscopies performed in the UK have been reported as occurring in the private sector),^31^ which could lead to unmeasured confounding. If the frequency of surveillance was greater among those with ‘high-risk’ polyps, this could lead to an overestimate or underestimate of the association between polyp characteristics and cancer risk (eg, from a greater detection of proximal colon cancer or removal of precancerous lesions, respectively). However, it was previously shown that before 2015 less than 2% of English UKFSST participants underwent colonoscopy within the BCSP, and after 2015, only 4% of these participants were still within the age criteria for further screening invitation or surveillance.^9^

The association between distal adenoma size or dysplasia and proximal colon cancer could potentially be explained by a greater likelihood of proximal SSLs, which are flat and less detectable with endoscopy,^32^ among participants with these distal adenoma characteristics detected. Although a previous study reported no associations between distal adenomas and the presence of SSLs, this was based on a small sample size.^33^ A lack of comprehensive recording of data on these lesions at the time of the UKFSST precluded further investigation of distal adenomas and proximal SSLs in current analyses.

Statistical models were adjusted for examination characteristics as these are known to impact adenoma detection rates,^34^,^36^ but residual confounding is possible due to observed differences in examination completeness between participants with ‘high-risk’ distal adenomas detected compared with those without.

The small numbers of participants with higher risk polyp characteristics (ie, multiple, larger size, villousness or high-grade dysplasia) may be explained by the inclusion of asymptomatic participants with no previous adenomas or endoscopy within 3 years prior to the study. As a result, the number of proximal colon cancer cases within some strata of exposure variables was low, resulting in wide 95% CIs. In addition, this study included men and women who registered to participate in a trial and, therefore, this population may have been more compliant with postscreening colonoscopy surveillance than the general UK population.

In summary, this study found that larger distal adenoma size and high-grade adenoma dysplasia at baseline were positively associated with long-term proximal colon cancer incidence. Distal findings could be important to predict those who are at higher risk of proximal colon cancer and require a baseline and/or surveillance colonoscopy to examine the proximal colon. Despite our observed higher risk of proximal colon cancer among those with small (6–9 mm) compared with diminutive adenomas (≤5 mm), small adenomas were not considered as ‘high-risk’ criteria for colonoscopy referral in the UKFSST or as stand-alone criteria for surveillance. However, the small number of cases in this study means that significant results (or a lack of association) should be interpreted with caution. Flexible sigmoidoscopy is no longer used for routine screening in the UK^37^; however, it is one of several screening tools used in the USA and Italy, and research is ongoing to assess its utility as a screening tool in Germany.^38^,^43^ The reduced cost and comparative ease of procedure make it a viable screening option. This study highlights the need for large, prospective studies with enough power to confirm our findings. For example, pooled analyses of polyp characteristics and proximal colon cancer from all four flexible sigmoidoscopy trials may provide more definitive conclusions.

## Supplementary material

10.1136/bmjgast-2025-001787 online supplemental file 1

10.1136/bmjgast-2025-001787 online supplemental file 2

## Data Availability

Data are available upon reasonable request.

## References

[1] Bray F, Laversanne M, Sung H (2024). Global cancer statistics 2022: GLOBOCAN estimates of incidence and mortality worldwide for 36 cancers in 185 countries. CA Cancer J Clin.

[2] Lam F, Laversanne M, Colombet M (2024). Global cancer observatory: cancer today. https://gco.iarc.who.int/today.

[3] Lin JS, Perdue LA, Henrikson NB (2021). Screening for Colorectal Cancer: Updated Evidence Report and Systematic Review for the US Preventive Services Task Force. J Am Med Assoc.

[4] Brenner H, Stock C, Hoffmeister M (2014). Effect of screening sigmoidoscopy and screening colonoscopy on colorectal cancer incidence and mortality: systematic review and meta-analysis of randomised controlled trials and observational studies. BMJ.

[5] Kanth P, Inadomi JM (2021). Screening and prevention of colorectal cancer. *BMJ*.

[6] Holme Ø, Løberg M, Kalager M (2018). Long-Term Effectiveness of Sigmoidoscopy Screening on Colorectal Cancer Incidence and Mortality in Women and Men: A Randomized Trial. Ann Intern Med.

[7] Senore C, Riggi E, Armaroli P (2022). Long-Term Follow-up of the Italian Flexible Sigmoidoscopy Screening Trial. Ann Intern Med.

[8] Miller EA, Pinsky PF, Schoen RE (2019). Effect of flexible sigmoidoscopy screening on colorectal cancer incidence and mortality: long-term follow-up of the randomised US PLCO cancer screening trial. Lancet Gastroenterol Hepatol.

[9] Wooldrage K, Robbins EC, Duffy SW (2024). Long-term effects of once-only flexible sigmoidoscopy screening on colorectal cancer incidence and mortality: 21-year follow-up of the UK Flexible Sigmoidoscopy Screening randomised controlled trial. Lancet Gastroenterol Hepatol.

[10] Dodou D, de Winter JCF (2012). The relationship between distal and proximal colonic neoplasia: a meta-analysis. J Gen Intern Med.

[11] Huang JLW, Wang YH, Jiang JY (2017). The Association between Distal Findings and Proximal Colorectal Neoplasia: A Systematic Review and Meta-Analysis. Am J Gastroenterol.

[12] Robbins EC, Wooldrage K, Saunders BP (2025). Long-term colorectal cancer incidence in a post-endoscopic screening cohort, accounting for surveillance, by baseline polyp group, anatomic subsite, and sex. J Med Screen.

[13] Rutter MD, East J, Rees CJ (2020). British Society of Gastroenterology/Association of Coloproctology of Great Britain and Ireland/Public Health England post-polypectomy and post-colorectal cancer resection surveillance guidelines. Gut.

[14] Cross A, Wooldrage K Multicentre randomised controlled trial of “once only” flexible sigmoidoscopy in prevention of bowel cancer morbidity and mortality. https://www.journalslibrary.nihr.ac.uk/programmes/hta/166501.

[15] Atkin W, Wooldrage K, Parkin DM (2017). Long term effects of once-only flexible sigmoidoscopy screening after 17 years of follow-up: the UK Flexible Sigmoidoscopy Screening randomised controlled trial. Lancet.

[16] Atkin WS, Cook CF, Cuzick J (2002). Single flexible sigmoidoscopy screening to prevent colorectal cancer: baseline findings of a UK multicentre randomised trial. Lancet.

[17] Atkin W, Rogers P, Cardwell C (2004). Wide variation in adenoma detection rates at screening flexible sigmoidoscopy. Gastroenterology.

[18] Atkin WS, Edwards R, Kralj-Hans I (2010). Once-only flexible sigmoidoscopy screening in prevention of colorectal cancer: a multicentre randomised controlled trial. Lancet.

[19] Jass JS, Sobin LH (1989). WHO international histological classification of tumours.

[20] World Health Organization (2015). International statistical classification of diseases and related health problems. 10th revision, fifth edition, 2016 ed.

[21] Percy C, Holten Vv, Muir CS (1990). International classification of diseases for oncology.

[22] StataCorp (2023). Stata Statistical Software: Release 18.

[23] Lewis JD, Ng K, Hung KE (2003). Detection of proximal adenomatous polyps with screening sigmoidoscopy: a systematic review and meta-analysis of screening colonoscopy. Arch Intern Med.

[24] Lin OS, Gerson LB, Soon M-S (2005). Risk of proximal colon neoplasia with distal hyperplastic polyps: a meta-analysis. Arch Intern Med.

[25] Gondal G, Grotmol T, Hofstad B (2003). Grading of distal colorectal adenomas as predictors for proximal colonic neoplasia and choice of endoscope in population screening: experience from the Norwegian Colorectal Cancer Prevention study (NORCCAP). Gut.

[26] Majumdar D, Bevan R, Essam M (2024). Adenoma characteristics in the English Bowel Cancer Screening Programme. Colorectal Dis.

[27] Risio M (2010). The natural history of adenomas (Reprinted from Best Practice & Research in Clinical Gastroenterology, vol 24, pg 271-280). Best Pract Res Cl Ga.

[28] Hall JF (2015). Management of Malignant Adenomas. Clin Colon Rectal Surg.

[29] Lee SP, Sung IK, Kim JH (2015). Risk factors for incomplete polyp resection during colonoscopic polypectomy. Gut Liver.

[30] Djinbachian R, Iratni R, Durand M (2020). Rates of Incomplete Resection of 1- to 20-mm Colorectal Polyps: A Systematic Review and Meta-Analysis. Gastroenterology.

[31] Beaton D, Sharp L, Trudgill N (2025). British Society of Gastroenterology national evaluation of colonoscopy quality: findings from the National Endoscopy Database. Gastrointest Endosc.

[32] East JE, Vieth M, Rex DK (2015). Serrated lesions in colorectal cancer screening: detection, resection, pathology and surveillance. Gut.

[33] Kahi CJ, Vemulapalli KC, Snover DC (2015). Findings in the distal colorectum are not associated with proximal advanced serrated lesions. Clin Gastroenterol Hepatol.

[34] Clark BT, Rustagi T, Laine L (2014). What level of bowel prep quality requires early repeat colonoscopy: systematic review and meta-analysis of the impact of preparation quality on adenoma detection rate. Am J Gastroenterol.

[35] Sulz MC, Kröger A, Prakash M (2016). Meta-Analysis of the Effect of Bowel Preparation on Adenoma Detection: Early Adenomas Affected Stronger than Advanced Adenomas. PLoS One.

[36] Bevan R, Blanks RG, Nickerson C (2019). Factors affecting adenoma detection rate in a national flexible sigmoidoscopy screening programme: a retrospective analysis. Lancet Gastroenterol Hepatol.

[37] Cancer Research (2021). Bowel scope screening to stop in England. https://news.cancerresearchuk.org/2021/01/14/bowel-scope-screening-to-stop-in-england/.

[38] Brinkmann M, Diedrich L, Krauth C (2021). General populations’ preferences for colorectal cancer screening: rationale and protocol for the discrete choice experiment in the SIGMO study. BMJ Open.

[39] Diedrich L, Brinkmann M, Dreier M (2022). Additional offer of sigmoidoscopy in colorectal cancer screening in Germany: rationale and protocol of the decision-analytic modelling approach in the SIGMO study. BMJ Open.

[40] US Preventive Services Task Force (2021). Screening for Colorectal Cancer: US Preventive Services Task Force Recommendation Statement. JAMA.

[41] Shaukat A, Kahi CJ, Burke CA (2021). ACG Clinical Guidelines: Colorectal Cancer Screening 2021. *Am J Gastroenterol*.

[42] Wolf AMD, Fontham ETH, Church TR (2018). Colorectal cancer screening for average-risk adults: 2018 guideline update from the American Cancer Society. CA Cancer J Clin.

[43] Zorzi M, Urso EDL (2023). Impact of colorectal cancer screening on incidence, mortality and surgery rates: Evidences from programs based on the fecal immunochemical test in Italy. Dig Liver Dis.

