# Supplementary material for: Association of distal adenoma and hyperplastic polyp characteristics with long-term proximal colon cancer risk: a secondary, observational analysis of data from the UK Flexible Sigmoidoscopy Screening Trial
Source: BMJ Open Gastroenterol. 2025 Jun 23;12(1):e001787. doi: 10.1136/bmjgast-2025-001787 (PMC12186034; doi:10.1136/bmjgast-2025-001787)
Supplement: online supplemental file 1 [file bmjgast-12-1-s001.pdf]

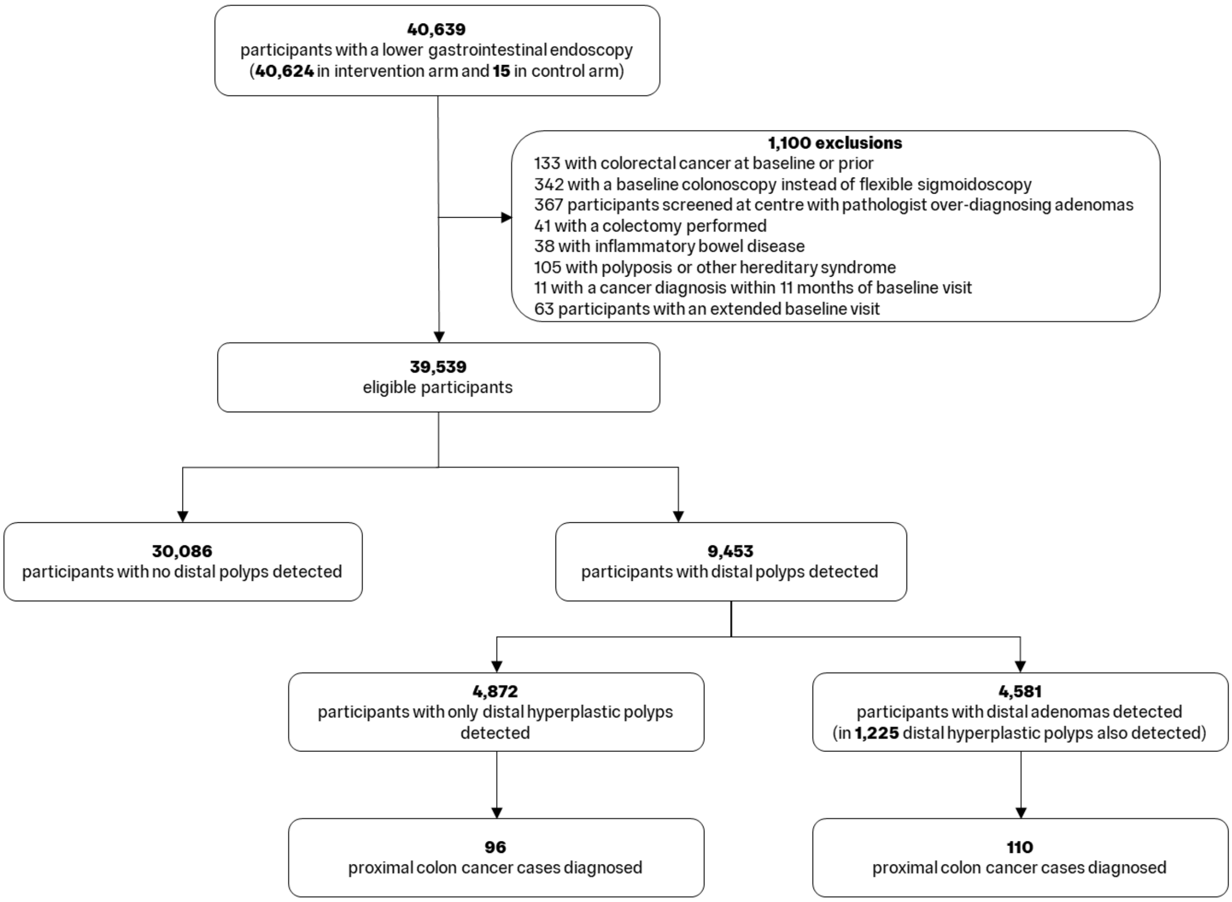

Supplemental figure 1: Participant flow diagram

Supplemental table 1: Description of participant demographics, examinations and surveillance visits by baseline distal polyp characteristics[a]

	Distal adenomas												Distal hyperplastic polyps					
	Number			Size (mm)			Histology			Dysplasia			Number			Size (mm)		
	1	2	≥3	≤5	6-9	≥10	Tubular	Tubulovillous /villous	Unknown	Low grade	High grade	Unknown	1	2	≥3	≤5	6-9	≥10
**N**	3,718 (81.2%)	667 (14.6%)	196 (4.3%)	2,464 (53.8%)	1,111 (24.3%)	1,006 (22.0%)	3,683 (80.4%)	836 (18.2%)	62 (1.4%)	4,339 (94.7%)	210 (4.6%)	32 (0.7%)	3,015 (61.9%)	975 (20.0%)	882 (18.1%)	4,320 (88.7%)	496 (10.2%)	56 (1.1%)
**Sex**																		
Women	1,344 (36.1%)	150 (22.5%)	32 (16.3%)	873 (35.4%)	365 (32.9%)	288 (28.6%)	1,229 (33.4%)	277 (33.1%)	20 (32.3%)	1,453 (33.5%)	62 (29.5%)	11 (34.4%)	1,287 (42.7%)	349 (35.8%)	311 (35.3%)	1,758 (40.7%)	170 (34.3%)	19 (33.9%)
Men	2,374 (63.9%)	517 (77.5%)	164 (83.7%)	1,591 (64.6%)	746 (67.1%)	718 (71.4%)	2,454 (66.6%)	559 (66.9%)	42 (67.7%)	2,886 (66.5%)	148 (70.5%)	21 (65.6%)	1,728 (57.3%)	626 (64.2%)	571 (64.7%)	2,562 (59.3%)	326 (65.7%)	37 (66.1%)
**Age group at randomisation (years)**																		
54-59	1,788 (48.1%)	284 (42.6%)	96 (49.0%)	1,211 (49.1%)	512 (46.1%)	445 (44.2%)	1,757 (47.7%)	382 (45.7%)	29 (46.8%)	2,051 (47.3%)	104 (49.5%)	13 (40.6%)	1,507 (50.0%)	512 (52.5%)	430 (48.8%)	2,168 (50.2%)	251 (50.6%)	30 (53.6%)
60-66	1,930 (51.9%)	383 (57.4%)	100 (51.0%)	1,253 (50.9%)	599 (53.9%)	561 (55.8%)	1,926 (52.3%)	454 (54.3%)	33 (53.2%)	2,288 (52.7%)	106 (50.5%)	19 (59.4%)	1,508 (50.0%)	463 (47.5%)	452 (51.2%)	2,152 (49.8%)	245 (49.4%)	26 (46.4%)
**Completeness of examination[b]**																		
Complete	3,519 (94.6%)	643 (96.4%)	195 (99.5%)	2,307 (93.6%)	1,067 (96.0%)	983 (97.7%)	3,476 (94.4%)	822 (98.3%)	59 (95.2%)	4,121 (95.0%)	205 (97.6%)	31 (96.9%)	2,698 (89.5%)	891 (91.4%)	809 (91.7%)	3,889 (90.0%)	455 (91.7%)	54 (96.4%)
Incomplete	171 (4.6%)	22 (3.3%)	1 (0.5%)	135 (5.5%)	36 (3.2%)	23 (2.3%)	177 (4.8%)	14 (1.7%)	3 (4.8%)	188 (4.3%)	5 (2.4%)	1 (3.1%)	281 (9.3%)	75 (7.7%)	59 (6.7%)	377 (8.7%)	36 (7.3%)	2 (3.6%)
Unknown	28 (0.8%)	2 (0.3%)	0 (0.0%)	22 (0.9%)	8 (0.7%)	0 (0.0%)	30 (0.8%)	0 (0.0%)	0 (0.0%)	30 (0.7%)	0 (0.0%)	0 (0.0%)	36 (1.2%)	9 (0.9%)	14 (1.6%)	54 (1.2%)	5 (1.0%)	0 (0.0%)
**Bowel preparation quality[b]**																		
Excellent or good	2,999 (80.7%)	547 (82.0%)	170 (86.7%)	1,906 (77.4%)	902 (81.2%)	908 (90.3%)	2,920 (79.3%)	745 (89.1%)	51 (82.3%)	3,501 (80.7%)	189 (90.0%)	26 (81.2%)	2,274 (75.4%)	694 (71.2%)	585 (66.3%)	3,159 (73.1%)	346 (69.8%)	48 (85.7%)
Adequate	550 (14.8%)	99 (14.8%)	22 (11.2%)	423 (17.2%)	162 (14.6%)	86 (8.5%)	582 (15.8%)	79 (9.4%)	10 (16.1%)	647 (14.9%)	19 (9.0%)	5 (15.6%)	593 (19.7%)	220 (22.6%)	242 (27.4%)	922 (21.3%)	127 (25.6%)	6 (10.7%)
Poor	36 (1.0%)	1 (0.1%)	2 (1.0%)	24 (1.0%)	5 (0.5%)	10 (1.0%)	31 (0.8%)	8 (1.0%)	0 (0.0%)	37 (0.9%)	2 (1.0%)	0 (0.0%)	37 (1.2%)	7 (0.7%)	9 (1.0%)	49 (1.1%)	2 (0.4%)	2 (3.6%)
Unknown	133 (3.6%)	20 (3.0%)	2 (1.0%)	111 (4.5%)	42 (3.8%)	2 (0.2%)	150 (4.1%)	4 (0.5%)	1 (1.6%)	154 (3.5%)	0 (0.0%)	1 (3.1%)	111 (3.7%)	54 (5.5%)	46 (5.2%)	190 (4.4%)	21 (4.2%)	0 (0.0%)
**Duration of baseline exam**																		
1 day	2,581 (69.4%)	316 (47.4%)	7 (3.6%)	2,117 (85.9%)	751 (67.6%)	36 (3.6%)	2,847 (77.3%)	27 (3.2%)	30 (48.4%)	2,892 (66.7%)	6 (2.9%)	6 (18.8%)	2,893 (96.0%)	923 (94.7%)	789 (89.5%)	4,155 (96.2%)	442 (89.1%)	8 (14.3%)
2-90 days	844 (22.7%)	232 (34.8%)	134 (68.4%)	255 (10.3%)	253 (22.8%)	702 (69.8%)	654 (17.8%)	532 (63.6%)	24 (38.7%)	1,049 (24.2%)	142 (67.6%)	19 (59.4%)	106 (3.5%)	40 (4.1%)	70 (7.9%)	141 (3.3%)	44 (8.9%)	31 (55.4%)
91-334 days	293 (7.9%)	119 (17.8%)	55 (28.1%)	92 (3.7%)	107 (9.6%)	268 (26.6%)	182 (4.9%)	277 (33.1%)	8 (12.9%)	398 (9.2%)	62 (29.5%)	7 (21.9%)	16 (0.5%)	12 (1.2%)	23 (2.6%)	24 (0.6%)	10 (2.0%)	17 (30.4%)
**Had a colonoscopy and/or surgery during baseline following flexible sigmoidoscopy**																		
Yes[c]	998 (26.8%)	332 (49.8%)	189 (96.4%)	247 (10.0%)	309 (27.8%)	963 (95.7%)	683 (18.5%)	807 (96.5%)	29 (46.8%)	1,293 (29.8%)	203 (96.7%)	23 (71.9%)	36 (1.2%)	17 (1.7%)	48 (5.4%)	28 (0.6%)	28 (5.6%)	45 (80.4%)
No	2,720 (73.2%)	335 (50.2%)	7 (3.6%)	2,217 (90.0%)	802 (72.2%)	43 (4.3%)	3,000 (81.5%)	29 (3.5%)	33 (53.2%)	3,046 (70.2%)	7 (3.3%)	9 (28.1%)	2,979 (98.8%)	958 (98.3%)	834 (94.6%)	4,292 (99.4%)	468 (94.4%)	11 (19.6%)

	Distal adenomas												Distal hyperplastic polyps					
	Number			Size (mm)			Histology			Dysplasia			Number			Size (mm)		
	1	2	≥3	≤5	6-9	≥10	Tubular	Tubulovillous /villous	Unknown	Low grade	High grade	Unknown	1	2	≥3	≤5	6-9	≥10
**Number of surveillance visits attended**																		
0	2,904	400	35	2,279	870	190	3,127	176	36	3,293	36	10	3,002	968	862	4,311	484	37
	(78.1%)	(60.0%)	(17.9%)	(92.5%)	(78.3%)	(18.9%)	(84.9%)	(21.1%)	(58.1%)	(75.9%)	(17.1%)	(31.2%)	(99.6%)	(99.3%)	(97.7%)	(99.8%)	(97.6%)	(66.1%)
1[d]	301	78	42	74	99	248	177	235	9	371	41	9	8	4	9	8	3	10
	(8.1%)	(11.7%)	(21.4%)	(3.0%)	(8.9%)	(24.7%)	(4.8%)	(28.1%)	(14.5%)	(8.6%)	(19.5%)	(28.1%)	(0.3%)	(0.4%)	(1.0%)	(0.2%)	(0.6%)	(17.9%)
≥2[e,f]	513	189	119	111	142	568	379	425	17	675	133	13	5	3	11	1	9	9
	(13.8%)	(28.3%)	(60.7%)	(4.5%)	(12.8%)	(56.5%)	(10.3%)	(50.8%)	(27.4%)	(15.6%)	(63.3%)	(40.6%)	(0.2%)	(0.3%)	(1.2%)	(0.0%)	(1.8%)	(16.1%)
**Hyperplastic polyps**																		
No	2,826	425	105	1,812	826	718	2,724	595	37	3,172	163	21	-		-	-		-
	(76.0%)	(63.7%)	(53.6%)	(73.5%)	(74.3%)	(71.4%)	(74.0%)	(71.2%)	(59.7%)	(73.1%)	(77.6%)	(65.6%)						
Yes	892	242	91	652	285	288	959	241	25	1,167	47	11	-		-	-		-
	(24.0%)	(36.3%)	(46.4%)	(26.5%)	(25.7%)	(28.6%)	(26.0%)	(28.8%)	(40.3%)	(26.9%)	(22.4%)	(34.4%)						

[a] Baseline distal adenoma and hyperplastic polyp characteristics defined using data from baseline flexible sigmoidoscopy examinations and data obtained on polyps detected between the rectum and the sigmoid colon during subsequent colonoscopy and surgery performed during a baseline visit.
[b] Completeness of examination and bowel preparation quality were defined using data from all examinations occurring during the baseline visit i.e., flexible sigmoidoscopy examinations and, where performed, subsequent colonoscopy and surgery during baseline.
[c] 1,385 (91%) of those participants with distal adenomas at baseline had high-risk distal adenoma findings.
[d] For participants with distal adenomas, the mean interval between baseline and the first surveillance visit was 2.8 years (standard deviation [sd] 1.2 years); for participants with distal hyperplastic polyps only, the mean interval between baseline and the first surveillance visit was 3.0 years (sd 1.4 years).
[e] For participants with distal adenomas, the mean interval between the first and second surveillance visits and the second and third surveillance visits was 3.6 years (sd 1.3 years) and 3.5 years (sd 1.4 years), respectively; for participants with distal hyperplastic polyps only, the mean interval between the first and second surveillance visits and the second and third surveillance visits was 3.3 years (sd 1.4 years) and 3.1 years (sd 1.2 years), respectively.
[f] For participants with distal adenomas, 11.3% had 2 surveillance visits, 4.9% had 3 surveillance visits and 1.7% had 4 or more surveillance visits.

Supplemental table 2: Number of cancers for strata of patient demographics, examinations and surveillance visits baseline distal adenoma and hyperplastic polyp characteristics[a].

	Distal adenomas													Distal hyperplastic polyps						
		Number			Size (mm)			Histology			Grade				Number			Size (mm)		
	All participants	1	2	≥3	≤5	6-9	≥10	Tubular	Tubulo-villous/ villous	Unknown	Low grade	High grade	Unknown	All participants	1	2	≥3	≤5	6-9	≥10
**N[b]**	110	84 (76.4%)	18 (16.4%)	8 (7.3%)	51 (46.4%)	34 (30.9%)	25 (22.7%)	91 (82.7%)	18 (16.4%)	1 (0.9%)	99 (90.0%)	10 (9.1%)	1 (0.9%)	96	57 (59.4%)	20 (20.8%)	19 (19.8%)	83 (86.5%)	11 (11.5%)	2 (2.1%)
**Sex**																				
Women	37	33	3	1	21	11	5	30	6	1	34	2	1	53	28	13	12	45	7	1
Men	73	51	15	7	30	23	20	61	12	0	65	8	0	43	29	7	7	38	4	1
**Age group at randomisation (years)**																				
54-59	47	40	4	3	24	12	11	38	9	0	41	6	0	43	28	7	8	38	5	0
60-66	63	44	14	5	27	22	14	53	9	1	58	4	1	53	29	13	11	45	6	2
**Completeness of examination[c]**																				
Complete	107	81	18	8	49	33	25	88	18	1	96	10	1	90	54	18	18	78	10	2
Incomplete	3	3	0	0	2	1	0	3	0	0	3	0	0	4	3	1	0	4	0	0
Unknown	-	-	-	-	-	-	-	-	-	-	-	-	-	2	0	1	1	1	1	0
**Bowel preparation quality[c]**																				
Excellent or good	92	70	15	7	41	29	22	75	16	1	81	10	1	79	46	17	16	67	10	2
Adequate	13	9	3	1	6	4	3	11	2	0	13	0	0	11	8	0	3	10	1	0
Poor	-	-	-	-	-	-	-	-	-	-	-	-	-	1	0	1	0	1	0	0
Unknown	5	5	0	0	4	1	0	5	0	0	5	0	0	5	3	2	0	5	0	0
**Duration of baseline exam**																				
1 day	76	67	9	0	48	27	1	73	2	1	75	0	1	90	56	18	16	80	9	1
2-90 days	24	12	7	5	2	5	17	14	10	0	18	6	0	4	0	2	2	1	2	1
91-334 days	10	5	2	3	1	2	7	4	6	0	6	4	0	2	1	0	1	2	0	0
**Had a colonoscopy and/or surgery during baseline following flexible sigmoidoscopy**																				
Yes	32	15	9	8	2	6	24	16	16	0	22	10	0	2	0	1	1	0	1	1
No	78	69	9	0	49	28	1	75	2	1	77	0	1	94	57	19	18	83	10	1
**Number of surveillance visits attended**																				
0	84	72	11	1	50	30	4	78	5	1	81	2	1	95	57	20	18	83	10	2
1[d]	3	3	0	0	1	1	1	1	2	0	2	1	0	-	-	-	-	-	-	-
>=2[e,f,g]	23	9	7	7	0	3	20	12	11	0	16	7	0	1	0	0	1	0	1	0
**Distal hyperplastic polyps**																				
No	73	59	8	6	28	28	17	59	13	1	63	9	1	-	-		-	-		-
Yes	37	25	10	2	23	6	8	32	5	0	36	1	0	-	-		-	-		-

[a] Baseline distal adenoma and hyperplastic polyp characteristics defined using data from baseline flexible sigmoidoscopy examinations and data obtained on polyps detected between the rectum and the sigmoid colon during subsequent colonoscopy and surgery performed during a baseline visit.
[b] Among participants with distal adenomas 42 of 110 (44%) proximal colon cancers and among those with distal hyperplastic polyps only, 38 of 96 (35%) proximal colon cancers were diagnosed after 2012 when no data were available on surveillance visits for UKFSST participants.
[c] Completeness of examination, bowel preparation was defined using data from all examinations occurring during the baseline visit i.e., flexible sigmoidoscopy examinations and, where performed, subsequent colonoscopy and surgery during baseline
[d] Among participants with distal adenomas detected who were diagnosed with proximal colon cancer the mean interval between baseline and the first surveillance visit was 2.6 years (standard deviation [sd] 1.1 years); not presented for those with distal hyperplastic polyps detected only as no participant was diagnosed with proximal colon cancer.
[e] For participants with distal adenomas the mean interval between the first and second surveillance visits and the second and third surveillance visits was 3.2 years (sd 1.1 years) and 2.5 years (sd 0.89 years) respectively; not presented for those with distal hyperplastic polyps detected only as there was only one participant diagnosed with proximal colon cancer.
[f] Among participants attending surveillance 6/26 proximal colon cancers were diagnosed during a surveillance visit; 9 cancers were diagnosed after 2012 during which time surveillance data were not available for this study. 5 of these 9 participants were ≥74 years (the age-limit for screening) in 2012.
[g] for participants with distal adenomas detected among these 23 participants diagnosed with cancer, 3 had 3 surveillance visits and 2 had 4 or more surveillance visits.

Supplemental table 3: Proximal colon polyp findings at baseline by distal adenoma characteristics among participants with 'high-risk'[a] adenomas findings at baseline who had a colonoscopy and/or surgery during baseline post flexible sigmoidoscopy.

		Distal adenoma characteristics											
	Total participants	Number			Size (mm)			Histology			Dysplasia		
		1	2	≥3	≤5	6-9	≥10	Tubular	Tubulovillous/ villous	Unknown	Low grade	High grade	Unknown
**Proximal colon findings at baseline**													
N	1,385	902 (65.1%)	294 (21.2%)	189 (13.6%)	189 (13.6%)	233 (16.8%)	963 (69.5%)	554 (40.0%)	807 (58.3%)	24 (1.7%)	1,161 (83.8%)	203 (14.7%)	21 (1.5%)
No hyperplastic polyps or adenomas	1,082 (78.1%)	744 (82.5%)	218 (74.1%)	120 (63.5%)	159 (84.1%)	186 (79.8%)	737 (76.5%)	423 (76.4%)	642 (79.6%)	17 (70.8%)	904 (77.9%)	161 (79.3%)	17 (81.0%)
Hyperplastic polyps only	43 (3.1%)	24 (2.7%)	9 (3.1%)	10 (5.3%)	4 (2.1%)	5 (2.1%)	34 (3.5%)	24 (4.3%)	17 (2.1%)	2 (8.3%)	35 (3.0%)	8 (3.9%)	0 (0.0%)
Non-high-risk adenoma finding[b]	172 (12.4%)	90 (10.0%)	40 (13.6%)	42 (22.2%)	20 (10.6%)	29 (12.4%)	123 (12.8%)	72 (13.0%)	96 (11.9%)	4 (16.7%)	151 (13.0%)	18 (8.9%)	3 (14.3%)
High-risk adenoma finding[a]	88 (6.4%)	44 (4.9%)	27 (9.2%)	17 (9.0%)	6 (3.2%)	13 (5.6%)	69 (7.2%)	35 (6.3%)	52 (6.4%)	1 (4.2%)	71 (6.1%)	16 (7.9%)	1 (4.8%)
**Participants with proximal adenomas at baseline**													
N	260												
**Adenoma number[c]**													
1	165 (63.5%)	93 (69.4%)	34 (50.7%)	38 (64.4%)	17 (65.4%)	28 (66.7%)	120 (62.5%)	62 (57.9%)	100 (67.6%)	3 (60.0%)	142 (64.0%)	20 (58.8%)	3 (75.0%)
2	50 (19.2%)	24 (17.9%)	16 (23.9%)	10 (16.9%)	6 (23.1%)	7 (16.7%)	37 (19.3%)	23 (21.5%)	25 (16.9%)	2 (40.0%)	42 (18.9%)	7 (20.6%)	1 (25.0%)
**≥3**	45 (17.3%)	17 (12.7%)	17 (25.4%)	11 (18.6%)	3 (11.5%)	7 (16.7%)	35 (18.2%)	22 (20.6%)	23 (15.5%)	0 (0.0%)	38 (17.1%)	7 (20.6%)	0 (0.0%)
**Adenoma size (mm)[c]**													
**≤5**	159 (61.2%)	88 (65.7%)	34 (50.7%)	37 (62.7%)	15 (57.7%)	23 (54.8%)	121 (63.0%)	78 (72.9%)	80 (54.1%)	1 (20.0%)	138 (62.2%)	20 (58.8%)	1 (25.0%)
6-9	60 (23.1%)	22 (16.4%)	24 (35.8%)	14 (23.7%)	8 (30.8%)	14 (33.3%)	38 (19.8%)	19 (17.8%)	38 (25.7%)	3 (60.0%)	54 (24.3%)	4 (11.8%)	2 (50.0%)
**≥10**	41 (15.8%)	24 (17.9%)	9 (13.4%)	8 (13.6%)	3 (11.5%)	5 (11.9%)	33 (17.2%)	10 (9.3%)	30 (20.3%)	1 (20.0%)	30 (13.5%)	10 (29.4%)	1 (25.0%)
**Adenoma histology[c]**													
Tubular	197 (75.8%)	100 (74.6%)	45 (67.2%)	52 (88.1%)	23 (88.5%)	33 (78.6%)	141 (73.4%)	91 (85.0%)	102 (68.9%)	4 (80.0%)	171 (77.0%)	23 (67.6%)	3 (75.0%)
Tubulovillous or villous	33 (12.7%)	16 (11.9%)	14 (20.9%)	3 (5.1%)	2 (7.7%)	4 (9.5%)	27 (14.1%)	8 (7.5%)	24 (16.2%)	1 (20.0%)	26 (11.7%)	6 (17.6%)	1 (25.0%)
Unknown	30 (11.5%)	18 (13.4%)	8 (11.9%)	4 (6.8%)	1 (3.8%)	5 (11.9%)	24 (12.5%)	8 (7.5%)	22 (14.9%)	0 (0.0%)	25 (11.3%)	5 (14.7%)	0 (0.0%)
**Adenoma dysplasia[c]**													
Low grade	227 (87.3%)	116 (86.6%)	56 (83.6%)	55 (93.2%)	25 (96.2%)	37 (88.1%)	165 (85.9%)	99 (92.5%)	124 (83.8%)	4 (80.0%)	197 (88.7%)	27 (79.4%)	3 (75.0%)
High grade	6 (2.3%)	2 (1.5%)	4 (6.0%)	0 (0.0%)	0 (0.0%)	0 (0.0%)	6 (3.1%)	1 (0.9%)	4 (2.7%)	1 (20.0%)	3 (1.4%)	2 (5.9%)	1 (25.0%)
Unknown	27 (10.4%)	16 (11.9%)	7 (10.4%)	4 (6.8%)	1 (3.8%)	5 (11.9%)	21 (10.9%)	7 (6.5%)	20 (13.5%)	0 (0.0%)	22 (9.9%)	5 (14.7%)	0 (0.0%)

[a] High-risk adenoma finding = ≥3 adenomas, adenomas ≥10mm, adenomas with tubulovillous or villous histology, adenomas with high-grade dysplasia
[b] Non-high-risk adenoma finding includes any adenoma not fitting the high-risk criteria
[c] Restricted to only those participants with proximal adenomas detected at baseline
